# Assessment of S100A8/A9 and resistin as predictive biomarkers for mortality in critically ill patients with sepsis

**DOI:** 10.3389/fcimb.2025.1555307

**Published:** 2025-06-03

**Authors:** Jing Chen, Zhengquan Liu, Fan Zhou, Ye Sun, Zhenyou Jiang, Pingsen Zhao

**Affiliations:** ^1^ Department of Laboratory Medicine, Yuebei People’s Hospital Affiliated to Shantou University Medical College, Shaoguan, China; ^2^ Laboratory for Diagnosis of Clinical Microbiology and Infection, Yuebei People’s Hospital Affiliated to Shantou University Medical College, Shaoguan, China; ^3^ Research Center for Interdisciplinary & High-quality Innovative Development in Laboratory Medicine, Shaoguan, China; ^4^ Shaoguan Municipal Quality Control Center for Laboratory Medicine, Yuebei People’s Hospital Affiliated to Shantou University Medical College, Shaoguan, China; ^5^ Shaoguan Municipal Quality Control Center for Surveillance of Bacterial Resistance, Yuebei People’s Hospital Affiliated to Shantou University Medical College, Shaoguan, China; ^6^ Shaoguan Engineering Research Center for Research and Development of Molecular and Cellular Technology in Rapid Diagnosis of Infectious Diseases and Cancer, Yuebei People’s Hospital Affiliated to Shantou University Medical College, Shaoguan, China; ^7^ Intensive Care Medicine Department, Yuebei People’s Hospital Affiliated to Shantou University Medical College, Shaoguan, China; ^8^ Key Laboratory of Viral Pathogenesis & Infection Prevention and Control (Jinan University), Ministry of Education, Guangzhou, China

**Keywords:** 28-day mortality, biomarkers, immunophenotype, resistin, S100A8/A9, sepsis

## Abstract

**Introduction:**

Sepsis is associated with high mortality. Early intervention is crucial to reducing sepsis-related mortality. This study aims to assess the clinical potential of S100A8/A9 and resistin as novel biomarkers for predicting mortality risk in sepsis patients.

**Method:**

Serum samples were collected and analyzed from 141 adult sepsis patients (discovery cohort), 43 non-sepsis intensive care units (ICU) patients, 15 healthy volunteers, and 55 sepsis patients along with 17 non-sepsis ICU patients (validation cohort). The 28-day mortality and sequential organ failure assessment (SOFA) scores of the participants were compared. Additionally, the predictive ability of S100A8/A9 and resistin for sepsis mortality was evaluated using the area under the receiver operating characteristic curve at ICU admission.

**Results:**

The concentrations of S100A8/A9 and resistin in sepsis patients were noticeably increased relative to non-sepsis patients and healthy controls. Serum S100A8/A9 concentrations in surviving sepsis patients were significantly higher than in non-surviving patients. On the day of admission, serum resistin concentrations in Gram-negative (G-) sepsis patients were considerably elevated relative to Gram-positive (G+) infected sepsis patients. Among sepsis patients admitted to the ICU, the AUC for S100A8/A9 in predicting 28-day mortality was 0.617 (*P* = 0.032; 95% confidence bounds 0.513–0.721), and for SOFA was 0.750 (*P* < 0.0001; 95% confidence bounds 0.660–0.840). Sepsis patients with high serum S100A8/A9 concentrations (≥ 377.53 ng/mL) had a higher survival rate relative to those with low concentrations (<377.53 ng/mL). In the validation cohort, the AUC for S100A8/A9 and 28-day mortality was 0.708 (*P* = 0.032; 95% confidence bounds 0.563–0.854), and for SOFA was 0.698 (*P* = 0.025; 95% confidence bounds 0.550–0.845). Additionally, sepsis patients with high serum S100A8/A9 concentrations (≥ 377.53 ng/mL) also had a higher survival rate relative to those with lower concentrations (< 377.53 ng/mL). Furthermore, serum resistin levels in patients with a normal phenotype and mixed phenotype with hyperinflammation were predictive of mortality, with an AUC of 0.810 (*P* = 0.034; 95% confidence bounds 0.605–1.00) and 0.708 (*P* = 0.015; 95% confidence bounds 0.571–0.846). In patients with a normal sepsis phenotype, those with high serum resistin levels (≥ 63.695 ng/mL) had a lower survival rate compared to those with low resistin levels (< 63.695 ng/mL). In contrast, in patients with a mixed phenotype with hyperinflammation, those with high serum resistin levels (≥ 107.64 ng/mL) had a higher survival rate compared to those with lower resistin levels (< 107.64 ng/mL).

**Discussion:**

Sepsis, the leading cause of death in intensive care unit patients. Identifying reliable biomarkers is essential for improving both the diagnosis and treatment of sepsis. We found that serum S100A8/A9 concentration at ICU admission is a significant predictor of 28-day mortality risk in sepsis patients. Additionally, resistin levels at ICU admission play an important role in predicting 28-day mortality risk in patients with both normal and mixed phenotypes with hyperinflammation. These findings suggest that S100A8/A9 and resistin could serve as effective biomarkers. Moreover, these findings could guide early clinical decisions in the treatment of sepsis patients.

## Graphical Abstract

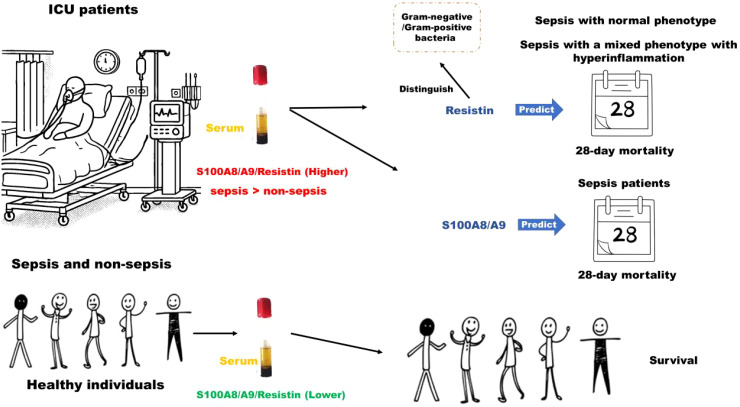

## Introduction

Sepsis is a life-threatening syndrome of organ dysfunction caused by a homeostatic failure in anti-pathogen defenses, with a mortality exceeding 25% (Seymour et al., 2016; Rhodes et al., 2017; Niederman et al., 2021). It poses a significant risk of death in intensive care units (ICUs) and has been recognized as a global health priority (Seymour et al., 2016; Rhodes et al., 2017; Niederman et al., 2021). Immune reaction indicators play critical roles in disease identification, timely recognition of multiorgan impairment, prognostic classification, clinical trajectory assessment, and therapeutic decision-making. Additionally, biomarkers can enhance clinical trials by identifying suitable patients and categorizing intervention risks (Hamilton et al., 2018; Barichello et al., 2022).

Researchers have been striving to identify more effective biomarkers for clinical sepsis patients, aiming to rapidly predict and assess sepsis prognosis and provide clearer treatment expectations to patients. The cytokine network plays a critical role in host defense, immune regulation, and the inflammatory response (Wu et al., 2021). Sepsis is a highly heterogeneous syndrome. This complexity has driven the emergence of endotype-driven approaches, which stratify septic patients through biomarker profiling, thereby facilitating tailored immunomodulatory interventions. Moreover, the simultaneous assessment of multiple biomarkers may address the limitations inherent in using a single biomarker for diagnosis or prognosis (Pierrakos et al., 2020).

Sepsis is characterized by cytokine-mediated excessive inflammation and a sustained decline in immune response. This process leads to the release of intracellular mediators known as damage-associated molecular patterns (DAMPs) (Lu et al., 2014; Wiersinga et al., 2014). Numerous DAMPs have been validated, some of which are currently used as inflammatory biomarkers (Lu et al., 2014; Wiersinga et al., 2014). Examples include proteins and cellular molecules associated with nucleic acids, such as heat shock proteins (HSPs), high mobility group protein 1 (HMGB-1), and members of the S100 family (Kataoka et al., 2014; Kigerl et al., 2014; Wiersinga et al., 2014). Recently, S100A8/A9 has emerged as a promising biomarker for sepsis, and it is a pathogenic molecule with both pro-inflammatory and immunosuppressive properties, primarily found in myeloid cells, including neutrophils and monocytes (Wang et al., 2023).

During sepsis, the S100A8/A9 complex activates the classical Toll-like receptor 4 (TLR4) signaling pathway in monocytes, inducing the expression of pro-inflammatory mediators related to NF-κB, and can be released as a DAMP (Odink et al., 1987; Lagasse and Weissman, 1992). Recent studies have also identified a distinct subpopulation of monocytes, referred to as low HLA-DR and high S100A monocytes. These cells are associated with the immunosuppressive state following sepsis. Inhibiting this subset of monocytes significantly improves sepsis-induced immunosuppression (Wang et al., 2023; Yao et al., 2023).

Resistin, another key pro-inflammatory cytokine (Bostrom et al., 2009; Johansson et al., 2009; Singbartl et al., 2016), plays a crucial role in metabolic inflammation and atherosclerosis, with human resistin expression increasing under inflammatory pathological conditions (Macdonald et al., 2014; Rachakonda et al., 2014; Macdonald et al., 2017). Moreover, growing evidence indicates that resistin serves as a key mediator in the immunosuppressive processes of sepsis (Koch et al., 2009; Vassiliadi et al., 2012; Macdonald et al., 2014; Miller et al., 2019).

Many potential biomarkers for sepsis have been proposed, but they have significant limitations in diagnosing the condition. Biomarkers such as C-reactive protein (CRP) and procalcitonin (PCT), which are the most widely used and studied, do not have the ability to predict mortality in sepsis patients (Pierrakos et al., 2020). Moreover, the Sepsis-3 consensus definition highlights that the role of biomarkers in diagnosing sepsis remains unclear (Singer et al., 2016). Additionally, growing evidence suggests that the morbidity and mortality associated with sepsis are increasingly linked to systemic immune dysfunction, including immunosuppression (Boomer et al., 2011; Hotchkiss et al., 2013). However, there are currently no effective markers to identify a patient’s immune status or predict mortality in sepsis patients with excessive inflammation or immunosuppression. Given that immune dysfunction biomarkers in sepsis are often associated with immune paralysis, relying on a single biomarker is not a completely reliable approach for guiding immune stimulation therapies. Measuring multiple biomarkers simultaneously may help address the limitations posed by any single biomarker (Pierrakos et al., 2020).

Early intervention is crucial to reducing sepsis-related mortality. Predicting mortality, assessing disease severity, guiding antibiotic use, and evaluating immune status in sepsis patients can enable timely proactive treatment, optimize therapeutic strategies, and improve patient outcomes, thereby increasing survival rates. Studies have demonstrated that both S100A8/A9 and resistin promote inflammatory responses by activating the TLR4 pathway and play key roles in immune suppression. Therefore, in this study, S100 family members S100A8/A9 and resistin were selected as biological targets. Preclinical identification of sepsis is critical. To this end, serum samples were collected from sepsis patients, non-sepsis patients on the day of admission, and healthy individuals. Along with factors such as CRP, PCT, IL-6, and white blood cell (WBC), we compared and analyzed the potential of resistin and S100A8/A9 as biomarkers for diagnosing sepsis, predicting mortality, generating survival curves, identifying Gram-negative bacteria (G-) and Gram-positive bacteria (G+), and immune subtype identification. A validation cohort was also included for further confirmation, with the aim of identifying new and effective targets for early sepsis intervention.

## Materials and methods

### Study population and parameters

On the day of ICU admission at Guangdong Yuebei People’s Hospital, 141 adult sepsis patients meeting the Sepsis-3 clinical criteria were enrolled in the discovery cohort (within 24h following ICU admission), whereas the validation cohort comprised 55 adult individuals with sepsis fulfilling identical inclusion criteria. After a 28-day follow-up period, patient survival during their ICU stay was recorded until discharge or death. In addition, 43 and 17 age-/gender-matched critically ill non-sepsis ICU patients were recruited as control groups for the discovery and validation cohorts, respectively. Including cases of burns, shock, road traffic injuries, consciousness disorders, falls, and major surgeries. Furthermore, healthy volunteers with 15 age/gender matching were also recruited from the physical examination center of Guangdong Yuebei People’s Hospital to serve as a healthy control group. A detailed flowchart of the study population is provided in **Supplementary Figures S1** and **S2**.

Inclusion criteria for patients with sepsis: (1) Patients must meet the Sepsis-3 diagnostic criteria, including confirmed or highly suspected infection, with a sequential organ failure assessment (SOFA) score ≥2 (Shankar-Hari et al., 2016; Singer et al., 2016); (2) Age ≥18 years; (3) Complete clinical records for all patients.

In addition to these diagnostic criteria, patients with septic shock must meet the following conditions: (1) Persistent hypotension requiring vasopressors to maintain a mean arterial pressure above 65 mmHg; or (2) Blood lactate levels exceeding 2 mmol/L in the absence of hypotension (6).

Exclusion criteria for sepsis patients: (1) patients re-admitted to the ICU; (2) Patients with severe liver or renal insufficiency, heart failure, cancer, acquired immunodeficiency syndrome (AIDS) or severe blood diseases; (3) Pregnant or postpartum patients.; (4) Patients with autoimmune diseases receiving immunosuppressant or glucocorticoid therapy; (5) Death within 24 hours of registration (6).

The experimental protocol was approved by the ethics committee of Guangdong Yuebei People’s Hospital, Shantou University School of Medicine, China (No. YBEC-KY(2021)-110), and informed consent was obtained from all participants or their legal representatives in accordance with the Declaration of Helsinki.

### Sample processing

Clinical parameters collected include WBC (BC-5800 automatic blood cell counter), PCT (Roche Cobas E801), CRP (Roche Cobas 8000), blood and bacterial cultures and other indicators. were measured on the day of enrollment, samples of blood were simultaneously collected, centrifuged at 350×g for 5 min within 30 min to isolate serum, aliquoted into 300 μL to minimize freeze-thaw degradation, and stored at -80°C (Sun S et al., 2021).

Samples underwent standardized preparation per the R&D Systems ELISA kit protocol prior to analytical procedures. Frozen specimens were gradually thawed at 4°C, followed by centrifugation to remove precipitates. Each analytical batch incorporated low, medium, and high concentration, and the calibration curve for ELISA demonstrated a coefficient of determination (R²) ≥ 0.99. Additionaly, hemolyzed samples were excluded. S100A8/A9 (R&D Systems, DS8900), resistin (R&D Systems, DRSN00), IL-6 (R&D Systems, D6050), interleukin-1β (IL-1β) (R&D Systems, DLB50), TNF-α (R&D Systems, QK225), interleukin-10 (IL-10) (R&D Systems, D1000B), and soluble programmed death-ligand 1 (sPD-L1) (Abcam, ab277712).

### Identification criteria for immune status of sepsis patients

To characterize inflammatory and immunosuppressive states in sepsis patients, we analyzed four pro-inflammatory cytokines (IL-6, IL-1β, TNF-α, and CRP) and two anti-inflammatory mediators (IL-10 and sPD-L1) (Zhou F et al., 2024). Biomarker reference thresholds were established using dual criteria: the 95th percentile of healthy volunteers and the 50th percentile (median) of septic patients in our cohort, with the higher value selected as the study-specific cut-off (Zhou F et al., 2024). Concentrations exceeding this threshold were defined as positive (+), while others as negative (-). Based on positivity counts, sepsis patients were categorized into five phenotypes: (1) Normal phenotype (negative for all cytokines), (2) Hyperinflammation only (≥ 1 pro-inflammatory positive, anti-inflammatory negative), (3) Immunosuppression only (pro-inflammatory negative, ≥ 1 anti-inflammatory positive), (4) Mixed phenotype with hyperinflammation (≥ 1 pro-inflammatory positive with single anti-inflammatory positivity), and (5) Mixed phenotype with Immunosuppression (≥ 1 pro-inflammatory positive with dual anti-inflammatory positivity) (Zhou F et al., 2024).

### Data processing and statistical methods

Statistical analyses and graph generation were conducted using GraphPad Prism (GraphPad Software 6.02) and IBM SPSS Statistics 26.0 (IBM Corp). Non normally distributed variables are represented by median (interquartile range), and comparison of skewed data between groups is conducted using non parametric Mann Whitney U test or one-way analysis of variance (Wu et al., 2021).To evaluate the predictive efficacy of S100A8/A9 for 28-day mortality, we constructed receiver operating characteristic (ROC) curves based on S100A8/A9 and resistin levels at admission, and calculated the area under the curve (AUC) with 95% confidence intervals. The 28 day cumulative mortality rate was analyzed using Kaplan Meier method with the log-rank *post hoc* test (Wu et al., 2021).The optimal cut-off point was determined by the Youden index, with sensitivity (SE) and specificity (SP) weighted accordingly (Wu et al., 2021). Additionally, survival curves for S100A8/A9 and resistin over 28 days were also plotted. Student’s t-test or the Mann-Whitney U test is used to compare normally distributed continuous variables, while chi-square test or Fisher’s exact probability method is used for categorical variables. Comparing multiple sets of quantitative data, choose one-way ANOVA or Kruskal-Wallis test based on distribution characteristics (Wu et al., 2021; Lan Y et al., 2024; Zhou F et al., 2024). All tests were bilateral, with a significance threshold set at *P <* 0.05 (Wu et al., 2021; Lan Y et al., 2024; Zhou F et al., 2024).

## Results

### Clinical characteristics of patients

141 adult sepsis patients, 43 non-septic ICU patients, and 15 healthy control volunteers were included in the discovery cohort. The validation cohort comprised 55 adult sepsis patients, 17 non-septic ICU patients. The basic demographic and clinical characteristics of these subjects are summarized in **Supplementary Table S1**. On the day of admission, non-survivors exhibited significantly higher acute physiological scores and SOFA scores, as well as a greater extent of organ damage compared to survivors. Additionally, sepsis patients had markedly increased median levels of CRP, PCT, and IL-6 compared to healthy controls (**Supplementary Figure S2A-C**).

### Serum levels of S100A8/A9 and resistin in sepsis patients

**Figure 1:**
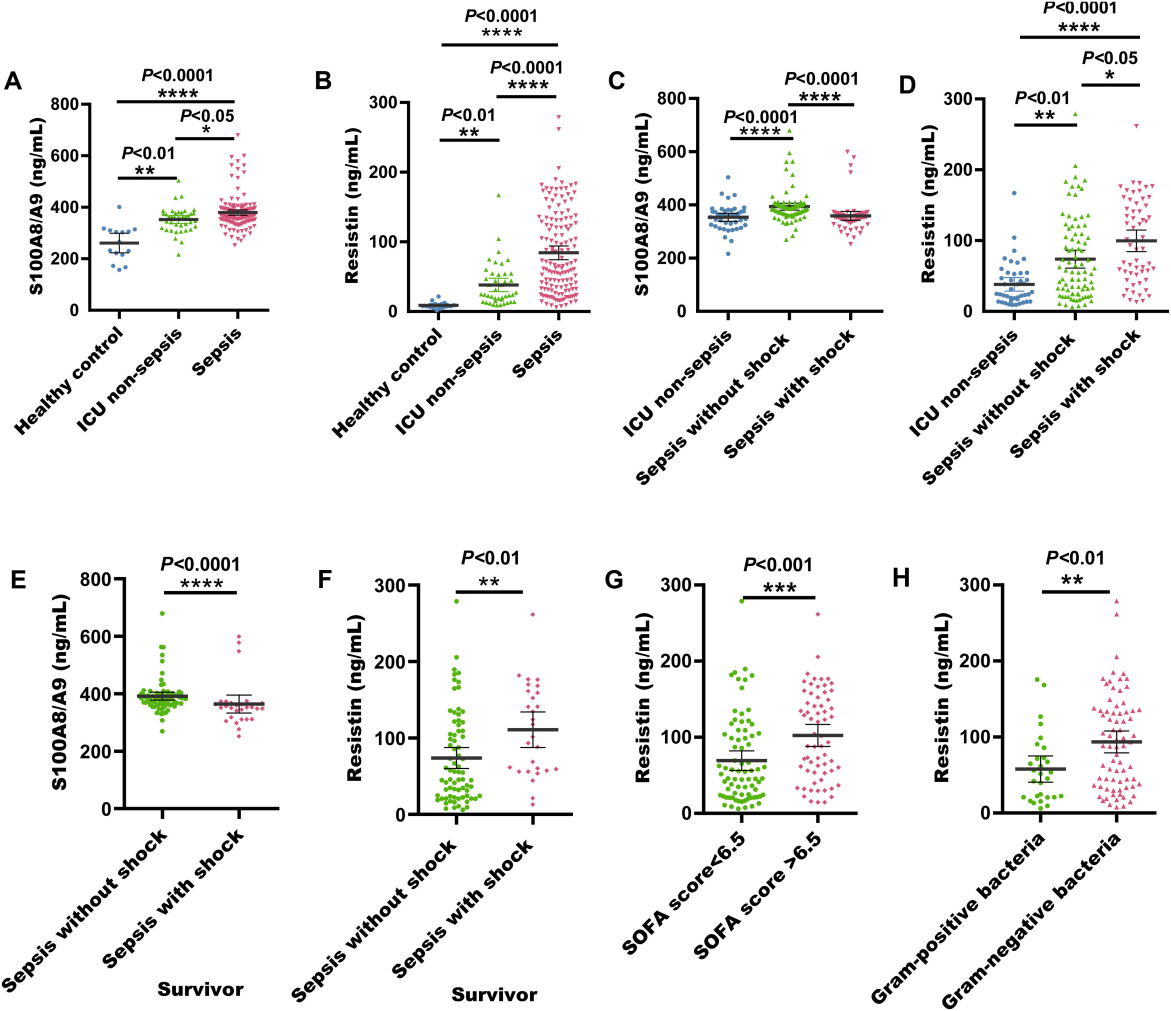
Serum S100A8/A9 and resistin levels at admission in the discovery cohort of adult sepsis patients. **(A)** S100A8/A9 concentrations in serum samples from healthy individuals, ICU non-sepsis patients, and sepsis patients. **(B)** Resistin concentrations in serum samples from healthy individuals, ICU non-sepsis patients, and sepsis patients. **(C)** S100A8/A9 concentrations in serum samples from patients with and without septic shock in the discovery cohort. **(D)** Resistin concentrations in serum samples from patients with and without septic shock in the discovery cohort. **(E)** S100A8/A9 concentrations in serum samples from sepsis with shock and sepsis without shock survivors. **(F)** Resistin concentrations in serum samples from sepsis with shock and sepsis without shock survivors. **(G)** Resistin concentrations in serum samples from sepsis patients with SOFA scores <6.5 and >6.5. **(H)** Resistin concentrations in serum samples from sepsis patients with G- and G+ bacterial infections. G+, Gram-positive; G-, Gram-negative; *P < 0.05, **P < 0.01, ***P < 0.001, ****P < 0.0001 (Kruskal-Wallis and Mann-Whitney U test).

On the day of admission, elevated serum concentrations of S100A8/A9 were observed in patients with sepsis. Meanwhile, the S100A8/A9 levels in non-septic ICU patients were noticeably increased relative to healthy standard group (**Figure 1A**). The median S100A8/A9 concentration at admission was 368.5 ng/mL (252.6-679.4 ng/mL) in sepsis patients, 356.8 ng/mL (216.1-503.9 ng/mL) in non-septic ICU patients, and 265.0 ng/mL (157.3-401.2 ng/mL) in healthy controls. Similarly, serum resistin concentrations were also substantially raised in sepsis patients in comparison with both non-septic ICU patients and healthy controls. And resistin concentrations in non-septic ICU patients were higher than healthy comparator group (**Figure 1B**). The median resistin concentration at admission was 66.93 ng/mL (6.15-278.9 ng/mL) in sepsis patients, 24.91 ng/mL (9.01-167.2 ng/mL) in non-septic ICU patients, and 7.63 ng/mL (1.93-21.68 ng/mL) in the healthy comparison group.

Among these 141 sepsis patients, serum S100A8/A9 concentrations were considerably elevated in those with non-shock sepsis (84 cases) relative to those with septic shock (*P* < 0.0001). In contrast, serum resistin concentrations were substantially reduced in non-shock sepsis patients (*P* < 0.05) (**Figures 1C, D**). Additionally, surviving patients with sepsis without shock demonstrated significantly elevated serum S100A8/A9 levels (*P* < 0.0001), whereas those with septic shock showed markedly increased resistin concentrations (*P* < 0.001) (**Figures 1E, F**). And nonsurvivors with sepsis without shock demonstrated significantly elevated serum S100A8/A9 levels (*P* < 0.001), whereas resistin levels remained comparable between septic shock and non-shock sepsis nonsurvivors (*P* = 0.38) (**Supplementary Figures S2D, E**). When stratified by a SOFA score threshold of 6.5, there was no difference in serum S100A8/A9 levels between patients with scores ≥ 6.5 and those with scores < 6.5 (*P* = 0.35) (**Supplementary Figure S2F**). However, patients with SOFA scores ≥ 6.5 had significantly elevated serum resistin concentrations (*P* < 0.01) (**Figure 1G**).

Among the 141 sepsis patients, 121 had confirmed bacterial infections, while 20 were diagnosed without bacterial infection. A total of 121 bacterial strains were isolated, including 29 G- bacteria, 75 G+ bacteria, 29 fungal strains, and 31 cases of mixed bacterial infections. The serum levels of S100A8/A9 and resistin were increased in sepsis patients upon admission. However, there was no noticeable difference in S100A8/A9 levels between patients with G- and G+ bacterial infections (*P* > 0.5) (**Supplementary Figure S2G**). In contrast, serum resistin levels were noticeably increased in patients with G- bacterial infections in comparison with those with gram-positive infections (*P* < 0.01) (**Figure 1H**).

### Correlation of S100A8/A9 and resistin with other biomolecules

**Figure 2:**
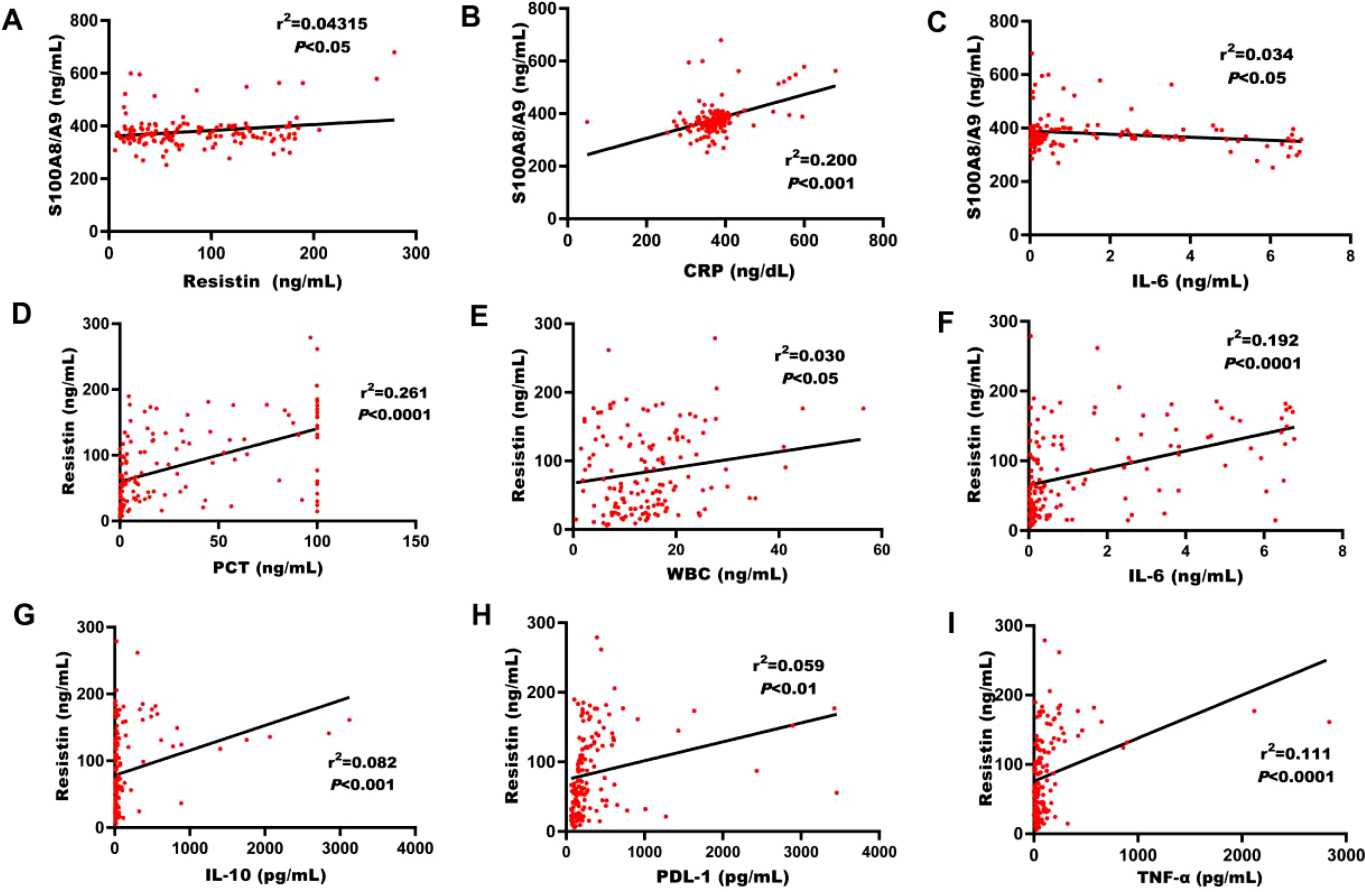
Correlation between S100A8/A9, resistin, and various biomarkers. **(A)** Correlation between S100A8/A9 levels and resistin in the discovery cohort of adult sepsis patients upon ICU admission. **(B)** Correlation of S100A8/A9 levels with CRP counts in the discovery cohort of adult patients with sepsis on ICU admission. **(C)** Correlation between S100A8/A9 levels and IL-6 in the discovery cohort. **(D)** Correlation between resistin levels and PCT in the discovery cohort. **(E)** Correlation between resistin levels and WBC in the discovery cohort. **(F)** Correlation between resistin levels and IL-6 in the discovery cohort. **(G)** Correlation between resistin levels and IL-10 in the discovery cohort. **(H)** Correlation between resistin levels and PDL-1 in the discovery cohort. **(I)** Correlation between resistin levels and TNF-α in the discovery cohort. CRP, C-reactive protein; IL-6, interleukin-6; IL-1β, interleukin-1β; TNF-α, tumor necrosis factor-alpha; IL-10, interleukin-10; PDL-1, programmed death-ligand-1. (Spearman's rank correlation coefficient).

S100A8/A9 showed a significant correlation with resistin (r² = 0.043, *P* < 0.05, **Figure 2A**). Additionally, S100A8/A9 was markedly correlated with CRP and the immune molecule IL-6 (*P* < 0.05, **Figures 2B, C**). Resistin was noticeably correlated with PCT (r² = 0.261, *P* < 0.0001), WBC (r² = 0.030, *P* < 0.05), IL-6 (r² = 0.192, *P* < 0.0001), IL-10 (r² = 0.082, *P* < 0.001), PDL-1 (r² = 0.059, *P* < 0.01), and TNF-α (r² = 0.111, *P* < 0.0001, **Figures 2D–I**).

### Prognostic value for 28-day survival in sepsis patients

**Figure 3:**
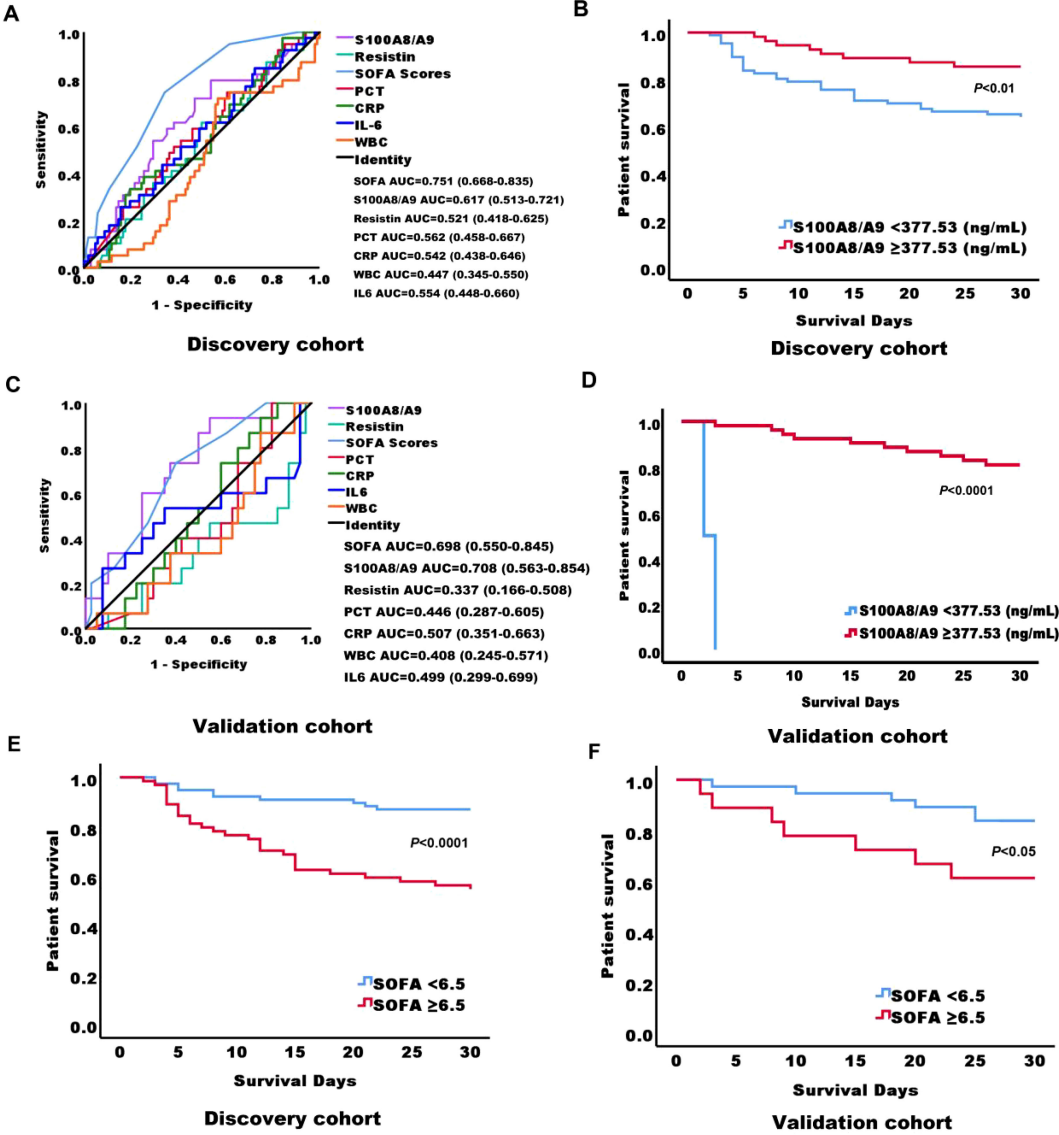
Receiver Operating Characteristic (ROC) curves and Kaplan-Meier survival curves for predicting 28-day mortality in adult sepsis patients based on serum biomarkers at admission. **(A)** ROC curves of serum biomarkers at admission in the discovery cohort of adult sepsis patients, predicting 28-day mortality. **(B)** Kaplan-Meier survival curves for 141 adult sepsis patients in the discovery cohort, stratified by S100A8/A9 levels using a cutoff of 377.53 ng/mL at ICU admission. **(C)** ROC curves of serum biomarkers at admission in the validation cohort of adult sepsis patients, predicting 28-day mortality. **(D)** Kaplan-Meier survival curves for 55 adult sepsis patients in the validation cohort, stratified by S100A8/A9 levels using a cutoff of 377.53 ng/mL at ICU admission. **(E)** Kaplan-Meier survival curves for 141 adult sepsis patients in the discovery cohort, stratified by SOFA scores using a cutoff of 6.5 at ICU admission. **(F)** Kaplan-Meier survival curves for 55 adult sepsis patients in the validation cohort, stratified by SOFA scores using a cutoff of 6.5 at ICU admission. AUC, area under the curve; S100A8/A9, S100 calcium-binding protein A8/A9; SOFA, sequential organ failure assessment; PCT, procalcitonin; CRP, C-reactive protein; IL-6, interleukin-6; WBC, white blood cell.

Among the 141 sepsis patients, the 28-day mortality rate was 27.7% (39/141). To identify better predictors of 28-day mortality, various parameters measured at admission were analyzed. The area under the curve (AUC) for SOFA, S100A8/A9, resistin, PCT, CRP, IL-6, and WBC in predicting 28-day mortality were 0.751 (*P* = 0.000, 95% confidence bounds 0.668–0.853), 0.617 (*P* = 0.034, 95% confidence bounds 0.513–0.721), 0.521 (*P* = 0.585, 95% confidence bounds 0.418–0.625), 0.562 (*P* = 0.225, 95% confidence bounds 0.458–0.667), 0.542 (*P* = 0.441, 95% confidence bounds 0.438–0.646), 0.554 (*P* = 0.321, 95% confidence bounds 0.448–0.660), and 0.447 (*P* = 0.335, 95% confidence bounds 0.345–0.550), respectively (**Figure 3A**; **Table 1**). The cut-off value for S100A8/A9 predicting 28-day mortality was 377.53 ng/mL. Kaplan-Meier survival analysis showed a substantial difference (*P* < 0.01) in survival between patients with high (≥ 377.53 ng/mL) and low S100A8/A9 levels. Patients with higher serum S100A8/A9 levels had a better survival rate than those with lower levels (**Figure 3B**).

**Table 1:** AUC, optimal cut-off points, validity indices, and predictive values of severity scores and biomarkers for 28-Day mortality in the discovery cohort.

Paramater	AUC (95% Cl)	Cut-off value	SE (%)	SP (%)	P-value	PPV (%)	NPV (%)
S100A8/A9	0.617 (0.513-0.721)	377.53	79.5	46.2	0.032	36.5	85.3
Resistin	0.521 (0.418-0.625)	62.01	61.5	50	0.697	32.4	76.9
SOFA	0.751 (0.668-0.835)	6.5	74.4	65.7	0.000	44.6	87.6
PCT	0.562 (0.458-0.667)	3.22	74.4	39.2	0.225	28.4	82.5
CRP	0.542 (0.438-0.646)	41.32	33.3	80.4	0.441	39.2	78.4
IL-6	0.554 (0.448-0.660)	0.109	84.6	27.5	0.321	29.8	82.3
WBC	0.447 (0.345-0.550)	11.16	71.8	43.1	0.335	30.0	79.4

AUC, area under the curve; S100A8/A9, S100 calcium-binding protein A8/A9; SOFA, sequential organ failure assessment; PCT, procalcitonin; CRP, C-reactive protein; IL-6, interleukin-6; WBC, white blood cell; Cut-off value the optimal value point with the highest sensitivity and specificity; SE, sensitivity; SP, Specificity; NPV, negative predictive value; PPV, positive predictive value.

In the validation cohort of 55 sepsis patients, the 28-day mortality rate was 27.7% (15/40). For this cohort, the AUC values for predicting 28-day mortality were 0.698 (*P* = 0.025, 95% confidence bounds 0.550–0.845) for SOFA, 0.708 (*P* = 0.032, 95% confidence bounds 0.563–0.854) for S100A8/A9, 0.337 (*P* = 0.064, 95% confidence bounds 0.166–0.508) for resistin, 0.446 (*P* = 0.539, 95% confidence bounds 0.287–0.605) for PCT, 0.507 (*P* = 0.940, 95% confidence bounds 0.458–0.667) for CRP, 0.499 (*P* = 0.321, 95% confidence bounds 0.299–0.699) for IL-6, and 0.468 (*P* =0.992, 95% confidence bounds 0.245–0.571) for WBC (**Figure 3C**; **Supplementary Table S3**). Consistent with the discovery cohort, in the validation cohort, survival analysis also revealed statistically significant variation (*P* < 0.0001) between patients with high (≥ 377.53 ng/mL) and low S100A8/A9 concentrations. As in the initial cohort, patients with higher serum S100A8/A9 levels demonstrated better survival outcomes (**Figure 3D**). Additionally, in the discovery cohort, the cut-off value for SOFA scores predicting 28-day mortality was 6.5. Kaplan-Meier survival analysis showed a substantial difference (*P* < 0.0001) in survival between patients with high (≥6.5) and low SOFA scores. Patients with higher serum SOFA scores had a lower survival rate than those with lower levels, and a consistent trend was observed in the validation cohort (**Figures 3E, F**).

### Serum expression levels of S100A8/A9 and resistin in sepsis patients across different immunotypes

**Figure 4:**
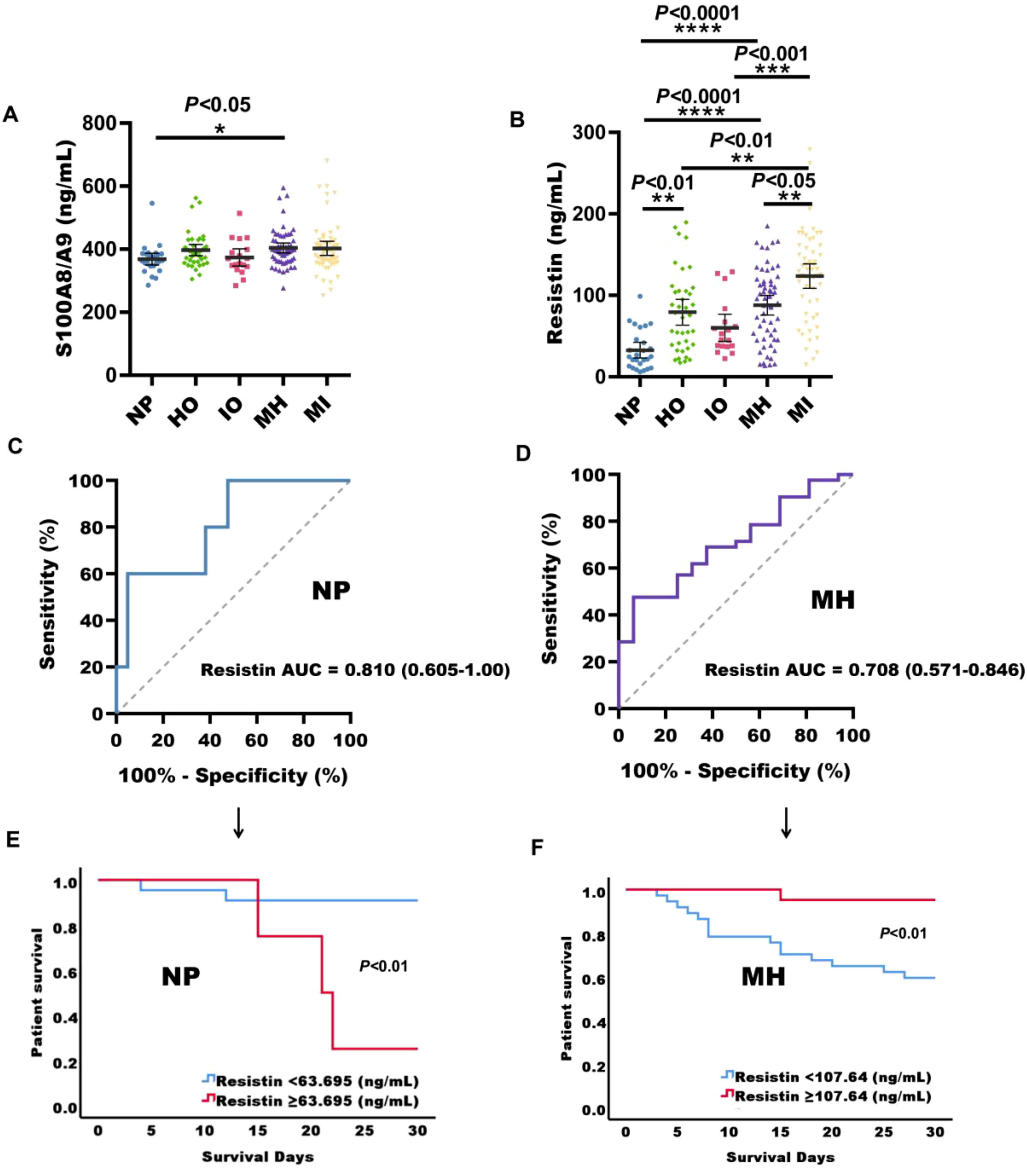
Serum S100A8/A9 and resistin expression levels in sepsis patients with different immunotypes. **(A)** S100A8/A9 concentrations in serum samples from sepsis patients with normal phenotype (NP), hyperinflammation only (HO), immunosuppression only (IO), mixed phenotype with hyperinflammation (MH), and mixed phenotype with immunosuppression (MI). **(B)** Resistin concentrations in serum samples from sepsis patients across the same phenotypes. **(C)** Receiver operating characteristic (ROC) curves for serum biomarkers at admission in predicting 28-day mortality in sepsis patients with normal phenotype. **(D)** ROC curves for serum biomarkers at admission in predicting 28-day mortality in sepsis patients with mixed phenotype and hyperinflammation. **(E)** Kaplan-Meier survival curves for 26 sepsis patients with normal phenotype based on the resistin cut-off (63.695 ng/mL) on ICU admission. **(F)** Kaplan-Meier survival curves for 64 sepsis patients with mixed phenotype with hyperinflammation based on the resistin cut-off (107.64 ng/mL) on ICU admission. NP, normal phenotype; HO, hyperinflammation only; IO, immunosuppression only; MH, mixed phenotype with hyperinflammation; MI, mixed phenotype with immunosuppression in sepsis patients; *P < 0.05, **P < 0.01, ***P < 0.001, ****P < 0.0001 (Kruskal-Wallis test).

A total of 196 sepsis patients were included in both the discovery and validation cohorts. Statistical analysis was performed on serum concentrations of CRP, IL-6, IL-1β, TNF-α, IL-10, and PDL1 from healthy individuals and sepsis patients, using a 95% CI. The highest values in each biomarker were selected as reference values. The reference values for CRP, IL-6, IL-1β, TNF-α, IL-10, and PDL1 were 19.51 ng/mL, 0.51 ng/mL, 27.75 pg/mL, 84.76 pg/mL, 22.11 pg/mL, and 288.55 pg/mL, respectively (**Supplementary Table S4**). Based on the classification criteria provided in **Supplementary Table S5**, patients were categorized into five groups: normal phenotype (26 cases), hyperinflammation only (39 cases), immunosuppression only (18 cases), mixed phenotype with hyperinflammation (58 cases), and mixed phenotype with immunosuppression (55 cases). A marked difference in S100A8/A9 expression levels was observed between patients with a normal phenotype and those with a mixed phenotype with hyperinflammation (*P* < 0.05), with the latter group exhibiting higher levels of S100A8/A9 (**Figure 4A**). In comparison with the normal phenotype, serum concentrations of resistin were significantly increased in patients with hyperinflammation only, immunosuppression only, mixed phenotype with hyperinflammation, and mixed phenotype with immunosuppression (*P* < 0.01) (**Figure 4B**). Among these groups, patients with a mixed phenotype with immunosuppression had the highest resistin levels, which were substantially higher than those in patients with a mixed phenotype with hyperinflammation (**Figure 4B**). Furthermore, serum resistin levels in patients with a normal phenotype and those with a mixed phenotype with hyperinflammation were predictive of mortality in this cohort (**Figures 4C, D**), with AUC of 0.810 (*P* = 0.034, 95% confidence bounds 0.605-1.00) and 0.708 (*P* = 0.015, 95% confidence bounds 0.571-0.846), respectively (**Supplementary Tables S6**, **S7**).

For sepsis patients with a normal phenotype, the resistin cut-off for predicting 28-day mortality was 63.695 ng/ml. Survival curve analysis revealed that patients with high resistin concentrations (≥ 63.695 ng/mL) had considerably decreased survival rates relative to those with lower resistin levels (*P* < 0.01). Conversely, in patients with a mixed phenotype with hyperinflammation, a higher resistin cut-off of 107.64 ng/ml was associated with improved survival (*P* < 0.01) (**Figures 4E, F**).

### Validation of cohort verification results

**Figure 5:**
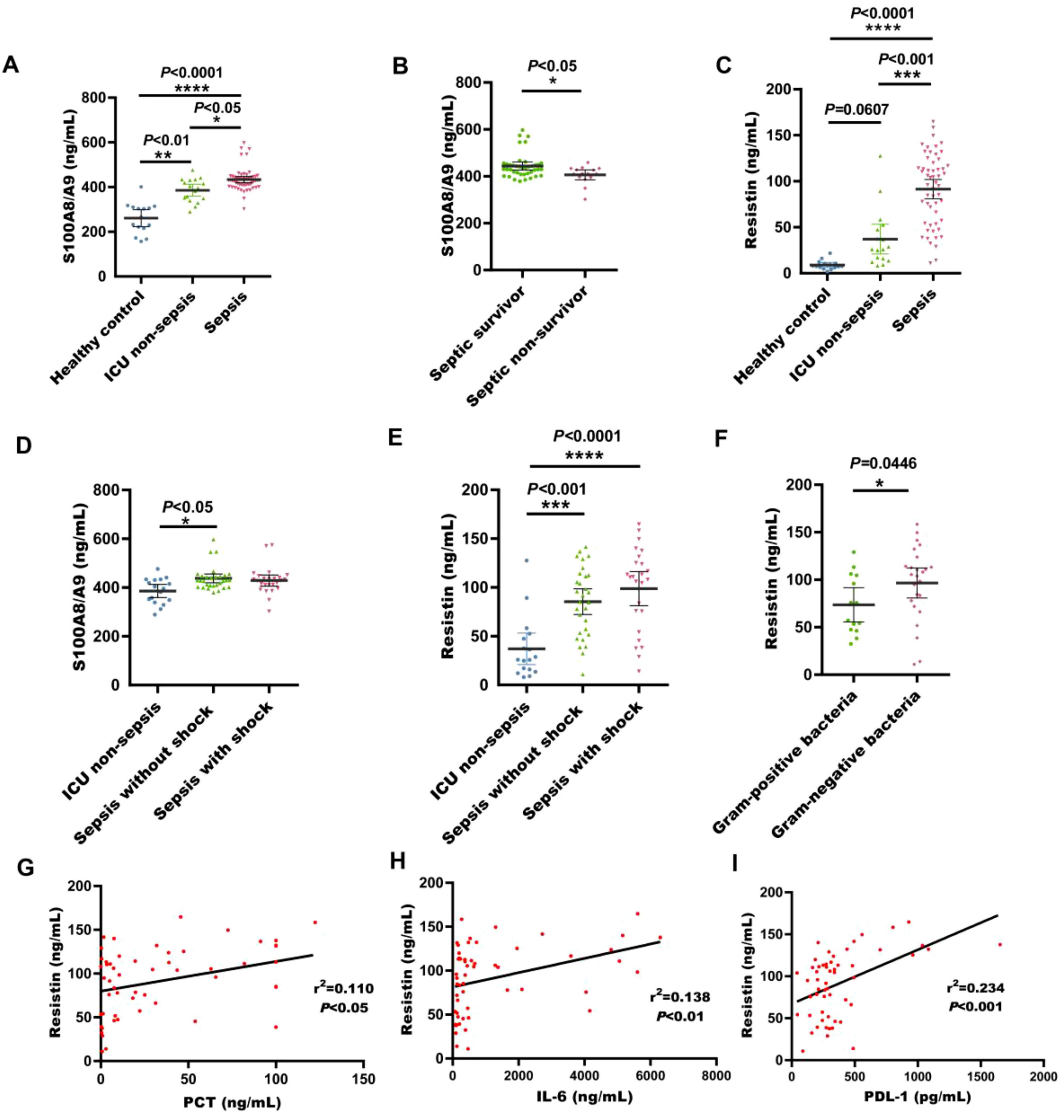
Serum S100A8/A9 and resisitin levels at admission were detected in the validation cohort of adult patients with sepsis. **(A)** S100A8/A9 concentrations in serum samples collected from healthy individuals, ICU non-sepsis patients and sepsis patients; **(B)** S100A8/A9 concentrations in serum samples collected from patients with septic shock and patients without shock in the validation cohort; **(C)** Resistin concentrations in serum samples collected from healthy individuals, ICU non-sepsis patients and sepsis patients; **(D)** S100A8/A9 concentrations in serum samples from patients with and without septic shock in the validation cohort. **(E)** Resistin concentrations in serum samples collected from patients with septic shock and patients without shock in the validation cohort; **(F)** Resistin concentrations in serum samples collected from patients with sepsis with G- bacteria and patients with sepsis with G+ bacteria; **(G)** Correlation of resistin levels with PCT counts in the validation cohort of adult patients with sepsis on ICU admission; **(H)** Correlation of resistin levels with IL-6 counts in the validation cohort of adult patients with sepsis on ICU admission; **(I)** Correlation of resistin levels with PDL-1 counts in the validation cohort of adult patients with sepsis on ICU admission; See **Figure 1** legend for expansion of abbreviation.*P < 0.05, **P < 0.01, ***P < 0.001, ****P < 0.0001 (Kruskal-Wallis and Mann-Whitney U test, Spearman's rank correlation coefficient.).

In the validation cohort, S100A8/A9 concentrations were substantially raised in sepsis patients in comparison with healthy individuals and non-sepsis patients (*P* < 0.05, **Figure 5A**). And it is highly expressed in surviving sepsis patients (*P* < 0.05, **Figure 5B**). Additionally, resistin levels were markedly increased in sepsis patients (*P* < 0.001, **Figure 5C**). The serum levels of S100A8/A9 in non-shock sepsis patients were also markedly higher than those in healthy individuals (*P* < 0.05, **Figure 5D**). Serum resistin concentrations in both non-shock and shock sepsis patients were noticeably increased than those in healthy individuals (*P* < 0.01, **Figure 5E**). Resistin expression was notably higher in patients with G- bacterial sepsis contrasted with G+ bacterial sepsis (*P* < 0.05, **Figure 5F**) Furthermore, resistin levels were significantly correlated with PCT, IL-6, and PDL1 (*P* < 0.05, **Figures 5G–I**).

## Discussion

Sepsis, the leading cause of death in intensive care unit patients, is triggered by an uncontrolled systemic response to infection, leading to widespread damage across multiple organs and systems. However, according to the Sepsis-3 consensus definition, the role of biomarkers in diagnosing sepsis remains unclear (Singer et al., 2016; Pierrakos et al., 2020). Identifying reliable biomarkers is essential for improving both the diagnosis and treatment of sepsis.

Both S100A8/A9 and resistin are known to promote inflammatory responses through activation of the TLR4 pathway and have been used as biomarkers for various inflammatory diseases (Donato et al., 2013; Yesudhas et al., 2014; Singbartl et al., 2016; Wang et al., 2023). We found that S100A8/A9 serves as a predictor of mortality in sepsis patients. First, serum S100A8/A9 levels were markedly elevated in sepsis patients relative to non-sepsis patients and healthy individuals, making it a potential diagnostic marker for sepsis (**Supplementary Figure S4**). Additionally, the serum concentration of S100A8/A9 in septic shock patients was notably lower than in those without shock. Furthermore, S100A8/A9 outperformed traditional biomarkers such as WBC, PCT, CRP, and IL-6 in predicting 28-day mortality at ICU admission. Survival curve analysis revealed that sepsis patients with lower serum S100A8/A9 levels at admission had reduced survival rates compared to those with higher levels. Importantly, the predictive value of S100A8/A9 for 28-day mortality was validated in an independent cohort of 55 sepsis patients. Although resistin did not predict mortality in sepsis patients, it effectively differentiated sepsis patients from non-sepsis patients and healthy individuals, as reflected by its increased serum levels in sepsis. This suggests its potential utility as a diagnostic marker. Moreover, resistin levels were noticeably increased in septic shock patients relative to those without shock, providing insight into the severity of sepsis.

Secondly, the serum S100A8/A9 level in patients with septic shock is significantly lower than that in patients without septic shock, including both survivor and nonsurvivor. In addition, S100A8/A9 outperformed WBC, PCT, CRP, and IL-6 in predicting 28-day mortality upon ICU admission. And according to survival curve analysis, patients with low serum S100A8/A9 admission levels in sepsis had lower survival rates than those with high serum S100A8/A9 admission levels. Most importantly, the role of S100A8/A9 in predicting 28-day mortality from sepsis was validated in an additional 55 adult patients with sepsis. Although resistin cannot predict the death of sepsis patients, it can still effectively distinguish sepsis patients from non-sepsis patients and healthy individuals. This is manifested by an increase in serum resistin expression concentration in sepsis patients, which can also serve as a diagnostic indicator for sepsis.

Additionally, the serum resistin levels in sepsis patients infected with G- and G+ bacteria were notably different. For instance, on the day of admission, resistin levels in patients with G- sepsis were considerably elevated than in those with G+ sepsis. Resistin can thus serve as a distinguishing marker between sepsis caused by G- and G+ infections. Sepsis is characterized by excessive inflammation and immunosuppression (Wu et al., 2021). Both S100A8/A9 and resistin levels were increased in patients with a mixed phenotype and high inflammation. Notably, resistin levels in patients with mixed phenotypes with immunosuppression were significantly higher than in those with mixed phenotypes with hyperinflammation.

Moreover, serum S100A8/A9 levels in sepsis patients show a positive correlation with serum resistin levels. S100A8/A9 is also positively correlated with CRP and the immune molecule IL-6, while resistin levels are positively correlated with PCT, WBC, IL-6, IL-10, PDL-1, and TNF-α. As highlighted, S100A8/A9 and resistin enhances the ability to distinguish sepsis patients from healthy individuals, assess the severity of sepsis, identify the infecting bacterial type, perform immunotyping of sepsis patients, and predict patient survival outcomes.

In conditions of widespread systemic inflammation, such as sepsis, neutrophils and monocytes serve as the immune system’s first line of defense against invading pathogens (Borregaard, 2010; Delano and Ward, 2016; Wang et al., 2023). The vesicle-dependent secretion pathway of S100A8/A9 is predominantly found in various immune cells, particularly macrophages and neutrophils. Additionally, inflammatory stimuli can trigger the passive release of S100A8/A9 from necrotic cells into the plasma, with circulating S100A8/A9 levels positively correlated with the extent of cell necrosis or tissue damage (Vogl et al., 2012; Sprenkeler et al., 2022). During sepsis, dysregulated pyroptosis leads to substantial S100A8/A9 release, exacerbating the inflammatory response and inducing platelet pyroptosis. As a result, serum S100A8/A9 concentrations are elevated in sepsis patients, consistent with previous findings (van Zoelen et al., 2009; Wirtz et al., 2020). Studies by have also demonstrated that higher levels of S100A8/A9 in the bloodstream can serve as an early indicator of sepsis and predict a higher risk of ICU admission. Furthermore, a significant positive correlation exists between elevated S100A8/A9 concentrations and poor clinical outcomes, including increased 28-day mortality in sepsis patients (Simm et al., 2016; Larsson et al., 2020). However, the results of our current study diverge from previous reports (Wirtz et al., 2020; Gao et al., 2022). While serum S100A8/A9 levels were higher in sepsis patients compared to non-sepsis patients and healthy individuals, severe sepsis cases, including non-survivors, exhibited lower S100A8/A9 expression levels than survivors. Notably, sepsis patients with low baseline calprotectin concentrations had significantly reduced survival rates (*P* < 0.01), contradicting some earlier findings (Wirtz et al., 2020; Gao et al., 2022).

The most likely explanation for this finding is that the majority of non-surviving sepsis patients in this study were in an immunosuppressive state, with compromised immune function, leading to decreased production and release of S100A8/A9. Previous studies have reported that many sepsis patients survive the initial hyperinflammatory phase but succumb to subsequent immunosuppression (Boomer et al., 2011; Hotchkiss et al., 2013). Traditionally, sepsis has been viewed as a two-stage process, beginning with an excessive inflammatory response followed by a compensatory anti-inflammatory phase. However, growing evidence suggests that immunosuppressive mechanisms may be active from the onset of sepsis (Liu D et al., 2022). This study found that while the serum concentration of S100A8/A9 in immunosuppressed patients at admission was not significantly different from that in patients with hyperinflammation—possibly due to the limited sample size after immune subtyping—the median levels showed a downward trend (**Figure 4A**). Moreover, patients with immunosuppressive sepsis exhibited a higher mortality rate compared to those with hyperinflammatory sepsis (**Supplementary Table S5**). This could provide a plausible explanation for the higher risk of death observed in sepsis patients with relatively low S100A8/A9 levels at hospital admission compared to those who survived.

Resistin has emerged as an important pro-inflammatory cytokine secreted by various cell types (Bostrom et al., 2009; Johansson et al., 2009; Singbartl et al., 2016). Its role as an acute-phase protein is closely linked to the severity of sepsis and septic shock, as well as to elevated levels of inflammatory cytokines, lactate, and serum creatinine (Koch et al., 2009; Vassiliadi et al., 2012; Macdonald et al., 2014). Studies have reported that serum resistin concentrations are significantly higher in patients with severe sepsis compared to those with milder forms of the condition (Singbartl et al., 2016; Lan Y et al., 2024), aligning with this study’s findings of increased resistin levels in patients with septic shock Additionally, consistent with the results of this study, Lehrke et al. observed that resistin levels in non-surviving patients were higher than in survivors, although the difference was not statistically significant (Lehrke et al., 2004). Sepsis results from a dysregulated host response to severe infection, with bacteria being the most common cause (Niederman et al., 2021). Rapid differentiation between G- and G+ pathogens is critical for guiding antibiotic therapy and improving patient outcomes. Currently, few biomarkers are recommended for distinguishing G-/G+ bacterial sepsis or improving sepsis prognosis (Huang et al., 2022). However, this study found that resistin expression levels were substantially raised in patients with Gram-negative sepsis, suggesting its potential use in identifying G-/G+ bacterial sepsis. Although resistin expression levels did not reach statistical significance in patients with excessive inflammation or immunosuppression, it was found that resistin levels were substantially raised in patients with inflammation only and mixed phenotype with immunosuppression compared to those with a normal phenotype. Interestingly, within the mixed phenotype group, patients with hyperinflammation had significantly lower resistin levels than those with immunosuppression. Resistin is a pro-inflammatory factor that is typically highly expressed during inflammation (Bostrom et al., 2009; Johansson et al., 2009; Singbartl et al., 2016). In our immune phenotyping results, the concentration of resistin was significantly higher in the inflammation-only compared to patients with normal phenotypes. Although no significant difference was observed between the inflammation-only and the immunosuppression-only - possibly due to the limited sample size, resistin still exhibited an upward trend in the former group. This is also evident from the significantly higher levels observed in patients with mixed inflammatory conditions compared to those with immunosuppression-only. Resistin is secreted not only by immune cells such as macrophages but also by adipocytes. In patients with mixed inflammation and immunosuppression, the anti-inflammatory/pro-inflammatory balance is severely disrupted, causing significant metabolic dysregulation, which may lead to increased adipocyte-derived resistin. Thus, the mixed immune group shows higher resistin levels than the inflammation-only group. Additionally, elevated resistin in mixed immunosuppression patients might further promote immune balance. However, these observations remain preliminary and necessitate additional experimental confirmation.

Notably, serum resistin concentrations in patients with both normal and mixed phenotypes with hyperinflammation were predictive of mortality. Additionally, this study demonstrated significant correlations between resistin, S100A8/A9, CRP, and IL-6 in sepsis patients. CRP is a well-established biomarker for sepsis, while IL-6, a proinflammatory factor, is strongly associated with disease severity and mortality in sepsis (Wu et al., 2021). Furthermore, resistin was significantly correlated with PD-L1 and TNF-α. PD-L1, an immune checkpoint molecule, suppresses T-cell activation by binding to its receptor PD-1, thereby reducing immune aggression and preventing tissue damage from excessive immune responses (Durrechou et al., 2020). TNF-α, a multifunctional cytokine primarily, plays a crucial role in inflammation, apoptosis, immune cell activation, and the acute phase response (Delgado and Brunner, 2019). The correlation of resistin with PD-L1 and TNF-α helps explain its elevated concentration in immunosuppressed patients. Additionally, resistin was significantly correlated with S100A8/A9, suggesting that the combination of these biomarkers could be useful for immune typing of sepsis patients. This approach not only facilitates targeted treatments based on immune profiles but also improves the prediction of mortality risk in sepsis patients.

Biomarkers have been extensively studied for their potential applications in sepsis patients, including diagnosis, prognosis, and guiding treatment decisions. However, the heterogeneity of sepsis at the individual level poses a significant challenge to the development of standardized care (Pierrakos et al., 2020). This complexity has spurred efforts to categorize patients into more homogeneous subgroups based on shared biological characteristics, which could facilitate the creation of tailored sepsis therapies (Liu D et al., 2022). Additionally, measuring multiple biomarkers simultaneously may help address the limitations of relying on any single biomarker. Currently, CRP and PCT are the most widely researched and utilized biomarkers in sepsis (Pierrakos et al., 2020). Both show transient increases during sepsis; however, they primarily reflect the intensity of inflammation and do not effectively predict mortality risk (Wu et al., 2021). In contrast, S100A8/A9 and resistin as sepsis biomarkers not only aids in diagnosing sepsis but also distinguishes between severe sepsis, G- and G+ infections, and different immune subtypes of sepsis patients. Furthermore, these markers can predict 28-day mortality in sepsis patients, particularly those with a normal phenotype or mixed phenotype with hyperinflammation.

While these findings provide valuable insights, our study has several limitations. First, it included only patients from North Guangdong People’s Hospital in Guangdong Province, China, which may limit the generalizability of the results. Further multi-center studies are necessary to account for regional and individual variability. Second, the study focused exclusively on adult sepsis patients. Additionally, we measured the serum levels of S100A8/A9 and resistin only on the first day of admission and assessed their association with short-term sepsis mortality risk. Expanding the observation period and evaluating long-term mortality risk would provide more comprehensive insights into sepsis prognosis. Moreover, as the clinical condition of sepsis patients can evolve and require varied supportive treatments, it remains unclear whether different treatment strategies may influence the study outcomes. Further research is needed to determine the impact of appropriate antibiotic therapy and other interventions on the daily serum levels of S100A8/A9 and resistin. In future studies, we plan to investigate the relationship between different treatment regimens and the dynamic changes in these biomarkers.

## Conclusion

Serum S100A8/A9 concentration at ICU admission is a significant predictor of 28-day mortality risk in sepsis patients. Additionally, resistin levels at ICU admission play an important role in predicting 28-day mortality risk in patients with both normal and mixed phenotypes with hyperinflammation. These findings suggest that S100A8/A9 and resistin could serve as effective biomarkers. Moreover, these findings could guide early clinical decisions in the treatment of sepsis patients.

## Data Availability

The original contributions presented in the study are included in the article/**Supplementary Material**. Further inquiries can be directed to the corresponding author.

## Supplementary material

Supplementary Figure 1 The flowchart of the study population. ROC, receiver operating characteristic.

Supplementary Figure 2 Serum biomarker levels at admission were measured in the discovery cohort of adult sepsis patients. **(A)** CRP concentrations in serum samples from healthy individuals, ICU non-sepsis patients, and sepsis patients. **(B)** PCT concentrations in serum samples from healthy individuals, ICU non-sepsis patients, and sepsis patients. **(C)** IL-6 concentrations in serum samples from healthy individuals, ICU non-sepsis patients, and sepsis patients. **(D)** S100A8/A9 concentrations in serum samples from sepsis with shock and sepsis without shock nonsurvivors. **(E)** Resistin concentrations in serum samples from sepsis with shock and sepsis without shock nonsurvivors. **(F)** S100A8/A9 concentrations in serum samples from adult sepsis patients with SOFA scores < 6.5 and > 6.5. **(G)** S100A8/A9 concentrations in serum samples from sepsis patients with Gram-positive and Gram-negative bacteria. CRP, C-reactive protein; IL-6, interleukin-6; IL-1β, interleukin-1β; **P* < 0.05, ***P* < 0.01, ****P* < 0.001, *****P* < 0.0001 (Kruskal-Wallis and Mann-Whitney U test).

Supplementary Figure 3 Receiver operating characteristic curves of serum biomarkers at admission in diagnosis of adult sepsis. **(A)** Receiver operating characteristic curves of serum biomarkers at admission in diagnosis of adult sepsis in discovery queue; **(B)** Receiver operating characteristic curves of serum biomarkers at admission in diagnosis of adult sepsis in validation queue. AUC, area under the curve; S100A8/A9, S100 calcium-binding protein A8/A9; SOFA, sequential organ failure assessment; PCT, procalcitonin; CRP, C-reactive protein; IL-6, interleukin-6; WBC, white blood cell.

## Glossary

AIDS: Acquired immunodeficiency syndrome
ANOVA: One-way analysis of variance
AUC: area under the curve
COPD: Chronic Obstructive Pulmonary Disease
CRP: C-reactive protein
DAMPs: Damage-associated molecular patterns
G-: Gram-negative bacteria
G+: Gram-positive bacteria
HBP: Heparin-Binding Protein
HLA-DR: Human leukocyte antigen DR
HMGB-1: High mobility group protein 1
HSPs: Heat shock proteins
ICU: Intensive care unit
IL-1β: Interleukin-1β
IL-6: Interleukin-6
IL-10: Interleukin-10
IQR: Inter quartile range
NPV: Negative predictive value
PCT: Procalcitonin
PDL-1: Programmed death-ligand-1
PPV: Positive predictive value
ROC: Receiver operating characteristic
SE: Sensitivity
SOFA: Sequential organ failure assessment
SP: Specificity
TLR4: Toll-like receptor 4
TNF-α: Tumor necrosis factor-alpha
WBC: White Blood Cell
sPD-L1: Soluble programmed death-ligand 1
95% CI: 95% confidence intervals
