# Supplementary figures and images for: Assessment of S100A8/A9 and resistin as predictive biomarkers for mortality in critically ill patients with sepsis
Source: Front Cell Infect Microbiol. 2025 Jun 3;15:1555307. doi: 10.3389/fcimb.2025.1555307 (PMC12188459; doi:10.3389/fcimb.2025.1555307)

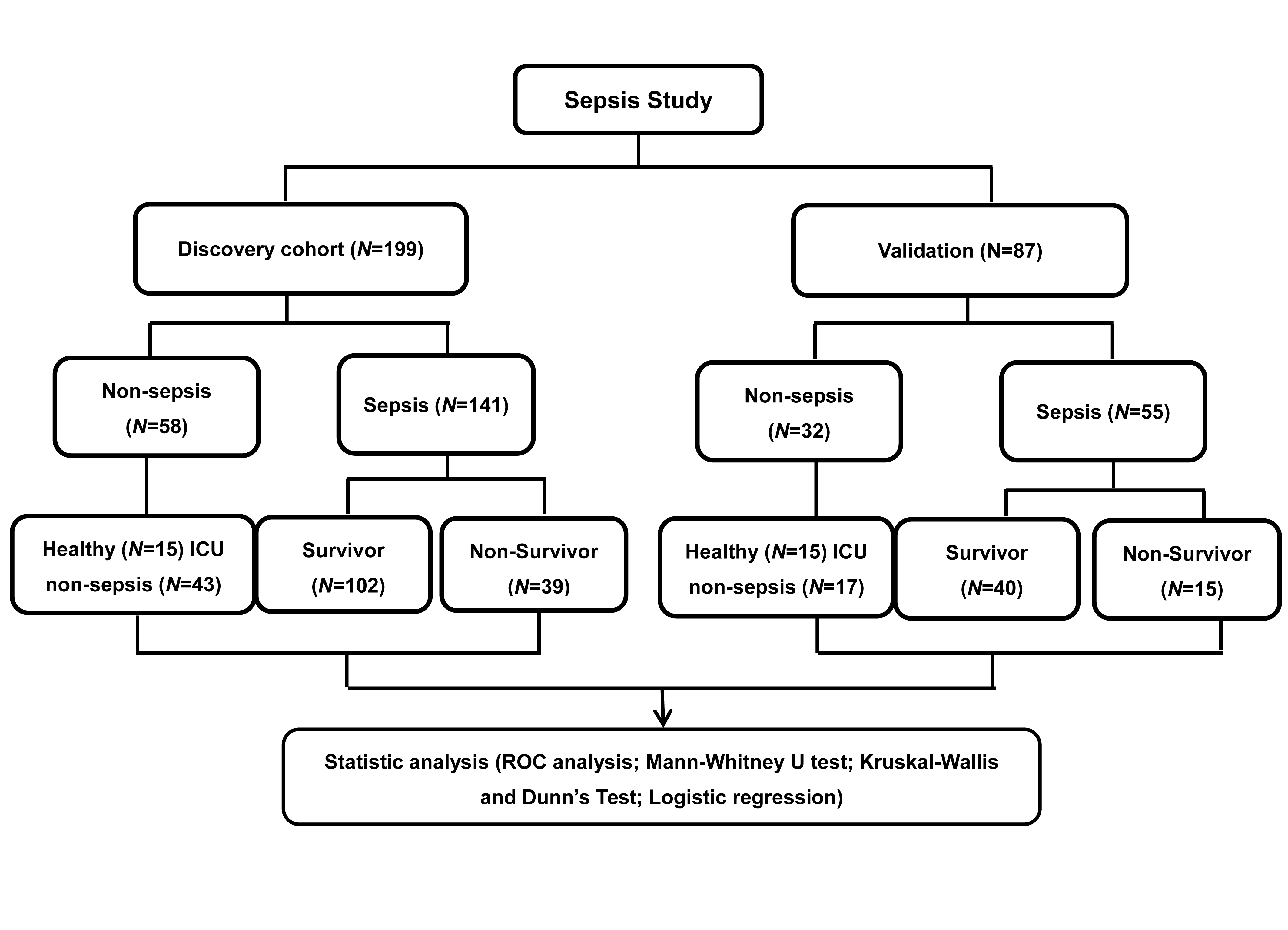

Supplement: Supplementary Figure 1 — The flowchart of the study population. ROC, receiver operating characteristic. [file Image1.tiff]

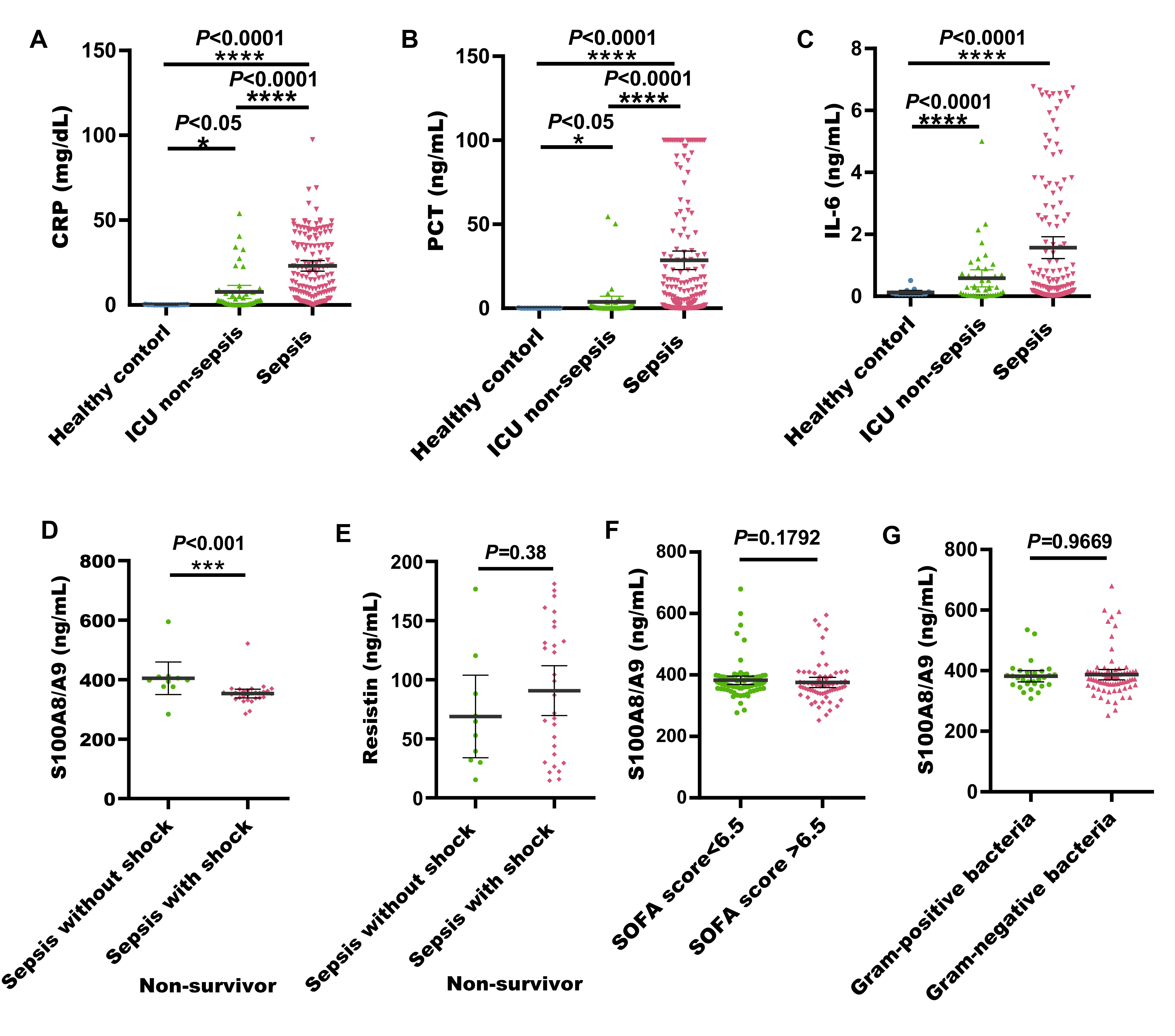

Supplement: Supplementary Figure 2 — Serum biomarker levels at admission were measured in the discovery cohort of adult sepsis patients. (A) CRP concentrations in serum samples from healthy individuals, ICU non-sepsis patients, and sepsis patients. (B) PCT concentrations in serum samples from healthy individuals, ICU non-sepsis patients, and sepsis patients. (C) IL-6 concentrations in serum samples from healthy individuals, ICU non-sepsis patients, and sepsis patients. (D) S100A8/A9 concentrations in serum samples from sepsis with shock and sepsis without shock nonsurvivors. (E) Resistin concentrations in serum samples from sepsis with shock and sepsis without shock nonsurvivors. (F) S100A8/A9 concentrations in serum samples from adult sepsis patients with SOFA scores < 6.5 and > 6.5. (G) S100A8/A9 concentrations in serum samples from sepsis patients with Gram-positive and Gram-negative bacteria. CRP, C-reactive protein; IL-6, interleukin-6; IL-1β, interleukin-1β; *P < 0.05, **P < 0.01, ***P < 0.001, ****P < 0.0001 (Kruskal-Wallis and Mann-Whitney U test). [file Image2.tif]

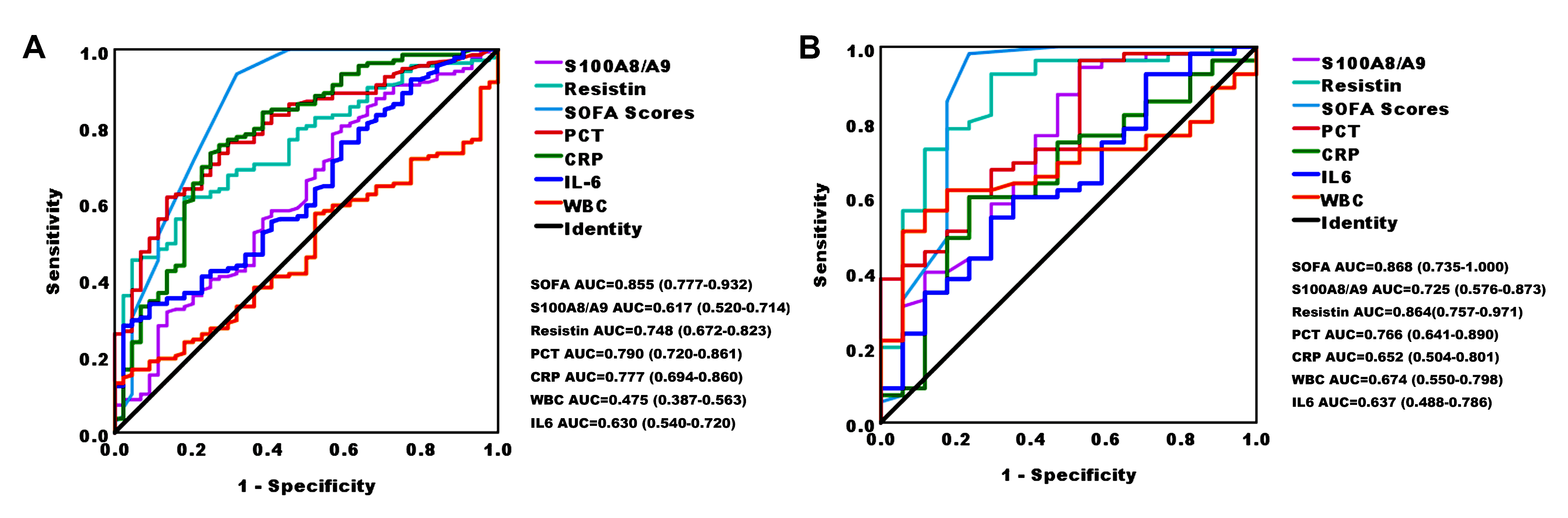

Supplement: Supplementary Figure 3 — Receiver operating characteristic curves of serum biomarkers at admission in diagnosis of adult sepsis. (A) Receiver operating characteristic curves of serum biomarkers at admission in diagnosis of adult sepsis in discovery queue; (B) Receiver operating characteristic curves of serum biomarkers at admission in diagnosis of adult sepsis in validation queue. AUC, area under the curve; S100A8/A9, S100 calcium-binding protein A8/A9; SOFA, sequential organ failure assessment; PCT, procalcitonin; CRP, C-reactive protein; IL-6, interleukin-6; WBC, white blood cell. [file Image3.tiff]
