# Supplementary material for: Assessment of S100A8/A9 and resistin as predictive biomarkers for mortality in critically ill patients with sepsis
Source: Front Cell Infect Microbiol. 2025 Jun 3;15:1555307. doi: 10.3389/fcimb.2025.1555307 (PMC12188459; doi:10.3389/fcimb.2025.1555307)
Supplement: Supplementary file 4 [file Table1.docx]

Table S1 Baseline characteristic of ICU patients and healthy volunteers.

| **Baseline**  **Characteristics** | **Healthy**  **Volunteers**  **n = 15** | **Sepsis**  **(Discovery queue)**  **n = 141** | **Non-sepsis**  **(Discovery queue) n = 43** | ***p* value** | **Sepsis**  **(Verify queue)**  **n = 55** | **Non-sepsis**  **(Verify queue) n = 17** | ***p* value** |
| --- | --- | --- | --- | --- | --- | --- | --- |
| Age, years (Mean ± SD) | 47.13 (± 15.05) | 63.41 ( ± 12.45) | 54.42 (± 12.10) | ns | 63.67 (± 13.39) | 45.53 (± 16.38) | ns |
| Sex, male (%) | 7 (46.67) | 87(61.70) | 23 (53.49) | ns | 30(54.55) | 14 (82.35) | ns |
| SOFA Scores, Median (IQR) | - | 6 (4.00-8.00) ^a^ | 2 (1.00-4.00) | - | 5 (4.00-7.00)^a^ | 1 (0.00-2.50) | - |
| **Laboratory values, median (IQR)** | | | | | | |  |
| CRP (mg/dL) | 0.11 (0.07-0.23) | 18.58 (6.72-39.13) ^a^ | 2.38 (0.32-8.18) | <0.0001 | 22.23 (8.38-40.00) | 8.86 (3.19-18.97) | <0.0001 |
| PCT (ng/mL) | 0.03 (0.03-0.05) | 12.60 (1.39-64.15) ^a^ | 0.38 (0.11-2.48) | <0.0001 | 18.30 (3.15-62.85) ^a^ | 4.120  (0.08-9.61) | <0.0001 |
| WBC (x 10^9^/L) | 6.94 (5.94-7.69) | 13.66 (8.13-12.20) | 14.83 (10.14-18.29) | <0.001 | 14.15 (9.55-19.51) ^a^ | 10.27 (9.20-10.98) | <0.0001 |
| IL-6 (ng/mL) | 37.13 (36.05-72.21) | 0.37  (0.12-2.60) | 0.28 (0.04-0.73) | <0.0001 | 0.37 (0.12-2.60) | 0.28 (0.04-0.73) | <0.0001 |
| HBP (ng/mL) | 1.15 (0.39-1.52) | 22.09 (12.30-35.87) | 29.42 (13.59-40.68) | <0.0001 | 11.43 (8.09-16.07) | 5.68  (4.83-6.21) | <0.0001 |
| **Comorbidities, n (%)** | | | | | | | |
| Hypertension | - | 49 (34.75) ^a^ | 11 (25.58) | - | 22 (40.00) ^a^ | 1 (5.88) | - |
| Diabetes | - | 48 (34.04) | 11 (25.58) | - | 15 (27.27) | 3 (17.65) | - |
| COPD | - | 16 (11.35) | 2 (4.65) | - | 4 (7.27) | 0 (0.00) | - |
| Liver disease | - | 38 (26.95) | 4 (9.30) | - | 15 (27.27) ^a^ | 0 (0.00) | - |
| Kidney disease | - | 25 (17.73) | 2 (4.65) | - | 14 (25.45) | 0 (0.00) | - |
| Cardiovascular disease | - | 43 (30.50) | 9 (20.93) | - | 18 (32.73) ^a^ | 1 (5.88) | - |
| **Outcome, n (%)** | | | | | | | |
| 28-day-Mortality | - | 39 (27.66) ^a^ | 6 (13.95) | - | 15 (27.27) ^a^ | 1 (5.88) | - |
| **Source of infection, n (%)** | | | | | | | |
| Respiratory tract | - | 64 (45.39) | - | - | 15 (27.27) | - | - |
| Urogenital | - | 36 (25.53) | - | - | 16 (29.09) | - | - |
| Abdominal | - | 42 (29.79) | - | - | 11 (20.00) | - | - |
| Skin or soft tissue | - | 7 (4.96) | - | - | 1 (1.81) | - | - |
| Surgical site | - | 0 (0.00) | - | - | 1 (1.81) | - | - |
| Blood culture positive | - | 66 (46.10) | - |  | 25 (45.45) | - | - |
| **Cultures, n (%)** | | | | | | | |
| Gram positive | - | 46 (32.62) | - |  | 18 (32.73) | - | - |
| Gram negative | - | 93 (65.96) | - |  | 29 (52.73) | - | - |
| Negative cultures | - | 10 (7.10) | - |  | 6 (10.91) | - | - |

*ICU, intensive care unit; IQR, inter quartile range; SOFA, sequential organ failure assessment;* *CRP, C-reactive protein; IL-6, interleukin-6; PCT, Procalcitonin; WBC, White Blood Cell; HBP, Heparin-Binding Protein ; COPD, Chronic Obstructive Pulmonary Disease; n (%): the count and percentage of cases; Significant differences between the variables were tested using Kruskal-Wallis (KW) test followed by Dunn’ spost hoc test or Mann-Whitney U test (MW) or Chi-square test; p* value, Sepsis vs Healthy; a, Sepsis vs Non-sepsis.

Table S2 Baseline characteristic of sepsis with shock and sepsis without shock.

| **Baseline**  **Characteristics** | **Sepsis with shock**  **(Discovery queue)**  **n = 57** | **Sepsis without shock**  **(Discovery queue) n = 84** | | ***p* value** | **Sepsis with shock**  **(Verify queue)**  **n = 25** | **Sepsis without shock**  **(Verify queue) n = 30** | ***p* value** |
| --- | --- | --- | --- | --- | --- | --- | --- |
| Age, years (Mean ± SD) | 66.02 ( ± 11.27) | 61.64 (± 12.79) | 0.04 | | 65.24 (± 15.21) | 62.37 (± 11.78) | 0.43 |
| Sex, male (%) | 35 (61.40) | 52.00 (61.90) | 0.83 | | 13(52.00) | 17 (56.67) | 0.73 |
| SOFA Scores, Median (IQR) | 8 (6.00-9.00) | 5 (4.00-7.00) | <0.0001 | | 6 (5.00-7.00)^a^ | 4.5 (3.75-6.25) | <0.01 |
| **Laboratory values, median (IQR)** | | | | | | |  |
| CRP (mg/dL) | 23.57 (9.02-43.06) | 15.60 (5.08-35.08) | 0.24 | | 15.43 (5.79-30.16) | 25.18 (9.97-43.14) | 0.43 |
| PCT (ng/mL) | 33.40 (3.86-93.95) | 4.79 (0.88-23.10) | <0.001 | | 7.60 (3.04-63.25) | 18.95  (4.25-63.54) | 0.84 |
| WBC (x 10^9^/L) | 13.50 (6.59-18.35) | 14.29 (8.93-19.21) | 0.25 | | 11.42 (6.94-18.84) | 15.31 (10.07-21.97) | 0.24 |
| IL-6 (ng/mL) | 0.59  (0.17-5.11) | 0.30 (0.18-2.12) | <0.01 | | 0.64 (0.19-4.10) | 0.38 (0.15-0.54) | 0.011 |
| HBP (ng/mL) | 25.73 (14.05-36.12) | 20.57 (10.25-35.63) | 0.29 | | 11.72 (8.05-13.77) | 10.68  (8.40-18.10) | 0.25 |
| **Comorbidities, n (%)** | | | | | | | |
| Hypertension | 19 (33.33) | 30 (35.71) | 0.77 | | 12 (48.00) | 10 (33.33) | 0.27 |
| Diabetes | 20 (35.09) | 28 (33.33) | 0.84 | | 7 (28.00) | 8 (26.67) | 0.81 |
| COPD | 5 (8.77) | 11 (13.09) | 0.30 | | 2 (8.00) | 2 (6.67) | 1.00 |
| Liver disease | 16 (26.95) | 22 (9.30) | 0.65 | | 8 (32.00) | 7 (23.33) | 0.58 |
| Kidney disease | 5 (8.77) | 20 (23.81) | 0.03 | | 7 (28.00) | 7 (23.33) | >0.99 |
| Cardiovascular disease | 21 (36.84) | 22 (26.19) | 0.27 | | 8 (32.00) | 10 (33.33) | 0.93 |
| **Outcome, n (%)** | | | | | | | |
| 28-day-Mortality | 29 (50.87) | 10 (11.90) | <0.001 | | 12 (48.00) | 3 (10.00) | <0.01 |
| **Source of infection, n (%)** | | | | | | | |
| Respiratory tract | 36 (63.16) | 28 (33.33) | <0.001 | | 7 (28.00) | 8 (26.67) | 0.81 |
| Urogenital | 13 (22.81) | 23 (27.38) | 0.527 | | 6 (24.00) | 10 (33.33) | 0.45 |
| Abdominal | 19 (33.33) | 23 (27.38) | 0.46 | | 4 (16.00) | 7 (23.33) | 0.56 |
| Skin or soft tissue | 2 (3.51) | 5 (5.95) | 0.68 | | 0 (0.00) | 1 (3.33) | >0.99 |
| Surgical site | 0 (0.00) | 0 (0.00) | >0.99 | | 1 (4.00) | 0 (0.00) | >0.99 |
| Blood culture positive | 28 (49.12) | 38 (45.24) | 0.65 | | 15 (60.00) | 10 (33.33) | 0.048 |
| **Cultures, n (%)** | | | | | | | |
| Gram positive | 25 (43.86) | 21 (25.00) | 0.03 | | 11 (44.00) | 7 (23.33) | 0.10 |
| Gram negative | 35 (61.40) | 58 (69.05) | 0.06 | | 13 (52.00) | 16 (53.33) | 0.92 |
| Negative cultures | 4 (7.02) | 6 (7.14) | 0.72 | | 3 (12.00) | 3 (10.00) | 1.00 |

*ICU, intensive care unit; IQR, inter quartile range; SOFA, sequential organ failure assessment;* *CRP, C-reactive protein; IL-6, interleukin-6; PCT, Procalcitonin; WBC, White Blood Cell; HBP, Heparin-Binding Protein ; COPD, Chronic Obstructive Pulmonary Disease; n (%): the count and percentage of cases; Significant differences between the variables were tested using Kruskal-Wallis (KW) test followed by Dunn’ spost hoc test or Mann-Whitney U test (MW) or Chi-square test; p* value, Sepsis vs Healthy; a, Sepsis vs Non-sepsis.

Table S3 AUC, optimal cut-off points, validity indices, and predictive values of severity scores and biomarkers for 28-Day mortality in the validation cohort.

| **Paramater** | **AUC (95% Cl)** | **Cut-off value** | **SE (%)** | **SP (%)** | ***P-*value** | **PPV (%)** | **NPV (%)** |
| --- | --- | --- | --- | --- | --- | --- | --- |
| S100A8/A9 | 0.708 (0.563-0.854) | 377.53 | 86 | 43 | 0.032 | 36.1 | 89.4 |
| Resistin | 0.337 (0.166-0.508) | 62.01 | 47.4 | 15.8 | 0.064 | 17.1 | 42.9 |
| SOFA | 0.698 (0.550-0.845) | 6.5 | 47.4 | 71.4 | 0.025 | 36.8 | 77.8 |
| PCT | 0.446 (0.287-0.605) | 3.22 | 71.9 | 28.1 | 0.539 | 27.3 | 72.7 |
| CRP | 0.507 (0.458-0.667) | 41.32 | 13.3 | 78.2 | 0.940 | 18.1 | 70.5 |
| IL-6 | 0.499 (0.299-0.699) | 0.109 | 86.6 | 26.5 | 0.321 | 30.4 | 83.2 |
| WBC | 0.468 (0.245-0.571) | 11.16 | 47.4 | 36.1 | 0.992 | 21.6 | 64.5 |

*See Table S1 legend for expansion of abbreviations.*

Table S4 Reference value for immunologic classification score.

| **Biomarkers** | **95% Cl of healthy individuals** | **Median of patients with sepsis** | **Reference value** |
| --- | --- | --- | --- |
| CRP (ng/dl) | 19.51 | 19.07 | 19.51 |
| IL-6 (ng/mL) | 0.51 | 0.38 | 0.51 |
| IL-1β (pg/mL) | 27.75 | 11.68 | 27.75 |
| TNF-α (pg/mL) | 84.76 | 60.71 | 84.76 |
| IL-10 (pg/mL) | 22.11 | 20.55 | 22.11 |
| PDL-1 (pg/mL) | 132.1 | 229.87 | 229.87 |

*CRP, C-reactive protein; IL-6,* *interleukin-6; IL-1β, interleukin-1β; TNF-α, tumor necrosis factor-alpha; IL-10, interleukin-10; PDL-1, programmed death-ligand-1.*

Table S5 lmunologic classification of patients with sepsis.

| **CRP, IL-6, IL-1β, TNFα** | **IL-10, PDL-1** | **Immunologic classicfication** | **Mortility numbers** |
| --- | --- | --- | --- |
| (-) | (-) | Normal phenotype | 5 |
| 1-4 (+) | (-) | Hyperinflammation only | 8 |
| (-) | 1-2 (+) | Immunosuppression only | 11 |
| 1-4 (+) | 1 (+) | Mixed phenotype with hyperinflammation | 16 |
| 1-4 (+) | 2 (+) | Mixed phenotype with Immunosuppression | 14 |

*CRP, C-reactive protein; IL-6, interleukin-6; IL-1β, interleukin-1β; TNF-α,* *tumor necrosis factor-alpha; IL-10, interleukin-10; PDL-1, programmed death-ligand-1.*

Table S6 AUC, optimal cut-off points, validity indices, and predictive values of severity scores and biomarkers for 28-Day mortality in the group of normal phenotype.

| **Paramater** | **AUC (95% Cl)** | **Cut-off value** | **SE (%)** | **SP (%)** | ***P-*value** | **PPV (%)** | **NPV (%)** |
| --- | --- | --- | --- | --- | --- | --- | --- |
| S100A8/A9 | 0.735 (0.478-0.932) | 379.23 | 57.1 | 57.1 | 0.162 | 24.0 | 84.9 |
| Resistin | 0.810 (0.605-1) | 63.695 | 60.0 | 95.2 | 0.034 | 71.2 | 88.9 |

*AUC, area under the curve; S100A8/A9, S100 calcium-binding protein A8/A9; Cut-off value the optimal value point with the highest sensitivity and specificity; SE, sensitivity; SP, Specificity; NPV, negative predictive value; PPV, positive predictive value.*

Table S7 AUC, optimal cut-off points, validity indices, and predictive values of severity scores and biomarkers for 28-Day mortality in the group of mixed phenotype with hyperinflammation.

| **Paramater** | **AUC (95% Cl)** | **Cut-off value** | **SE (%)** | **SP (%)** | ***P-*value** | **PPV (%)** | **NPV (%)** |
| --- | --- | --- | --- | --- | --- | --- | --- |
| S100A8/A9 | 0.518 (0.345-0.690) | 399.37 | 62.5 | 50 | 0.835 | 32.3 | 77.8 |
| Resistin | 0.708 (0.571-0.846) | 107.64 | 93.8 | 47.6 | 0.015 | 40.6 | 95.37 |

*See Table 7 legend for expansion of abbreviations.*
